# Joint Optimization of Control Strategy and Energy Consumption for Energy Harvesting WSAN

**DOI:** 10.3390/e24050723

**Published:** 2022-05-19

**Authors:** Zhuwei Wang, Zhicheng Liu, Lihan Liu, Chao Fang, Meng Li, Jingcheng Zhao

**Affiliations:** 1 Faculty of Information Technology, Beijing University of Technology, Beijing 100124, China; wangzhuwei@bjut.edu.cn (Z.W.); liuzc2020@emails.bjut.edu.cn (Z.L.); fangchao@bjut.edu.cn (C.F.); limeng720@bjut.edu.cn (M.L.); 2 School of Information, Beijing Wuzi University, Beijing 101149, China; 3 Beijing Academy of Science and Technology, Beijing 100089, China; zhaojingcheng@bjss.org.cn

**Keywords:** energy efficiency, energy harvesting controller, network-induced delay, UAV formation application

## Abstract

With the rapid development of wireless sensor technology, recent progress in wireless sensor and actuator networks (WSANs) with energy harvesting provide the possibility for various real-time applications. Meanwhile, extensive research activities are carried out in the fields of efficient energy allocation and control strategy design. However, the joint design considering physical plant control, energy harvesting, and consumption is rarely concerned in existing works. In this paper, in order to enhance system control stability and promote quality of service for the WSAN energy efficiency, a novel three-step joint optimization algorithm is proposed through control strategy and energy management analysis. First, the optimal sampling interval can be obtained based on energy harvesting, consumption, and remaining conditions. Then, the control gain for each sampling interval is derived by using a backward iteration. Finally, the optimal control strategy is determined as a linear function of the current plant states and previous control strategies. The application of UAV formation flight system demonstrates that better system performance and control stability can be achieved by the proposed joint optimization design for all poor, sufficient, and general energy harvesting scenarios.

## 1. Introduction

Wireless sensor and actuator network (WSAN), typically consisting of sensors, controllers, and actuators is one of the most critical wireless communication applications [1]. With the characteristic of spatially distributed nodes, WSANs are able to sense, communicate, compute, and cache to meet the demands of both high reliability and low latency. Efficient information sharing and energy consumption management can be achieved in the closed-loop feedback control network through proper resource allocation and control strategy design. Currently, WSAN has already become an attractive research topic in various application areas such as Internet of Thing (IoT), connected vehicle systems, smart cities, and smart grids [2,3,4].

WSAN takes advantage of wireless networks to provide information sharing, resource utilization, and plant control. However, there are still some challenges introduced, especially with the increasing number of connected devices and sensor nodes [5,6]. One of the problems is the control strategy design integrating with the inherent features of wireless networks [7]. In general, wireless communications within the WSANs introduced wireless communication features such as network-induced delays, packet losses, and disturbances. However, lots of research always focus on perfect system conditions. Actually, issues such as communication delays can generally lead to performance degradation or even instability of the closed-loop system. Therefore, the optimal control design to ensure the feasibility and efficiency of the WSAN system subject to imperfect wireless features has been extensively concerned [8]. In addition, the fixed sampling interval will result in low energy utilization efficiency or failure to obtain high-quality state samplings when the plant is in high mobility. Therefore, it is desirable to provide an adaptive sampling interval design to improve energy utilization and system stability [9].

In addition, energy management has gradually become another challenging problem due to the limitation on battery-powered sensor nodes in WSANs. Over the years, researchers have focused on energy-saving techniques to minimize the energy consumption of sensor nodes related to the medium access control, duty cycle, and routing design [10]. However, the lifetime of sensor nodes is still limited. Once the battery is used up, the sensor nodes will no longer participate in the operation of the network. Currently, energy harvesting, as a new technology that can collect external energy, has become a potential technology to address energy restriction issues in WSANs [11]. However, restricted by the limited capacity of the equipped energy buffer, how to effectively store and utilize the harvested energy is still a great challenge [12].

However, most of the existing articles focus on either control strategy design or energy harvesting and consumption in WSAN. The overview articles [13] and our earlier work [14] reveal the potential benefits of jointly optimizing control strategy and energy consumption. However, the collaborative optimization of the energy management and the control strategy with energy harvesting capacity has not been studied sufficiently. In this paper, a novel framework to jointly consider network-induced delay, adaptive sampling intervals, and energy management is proposed to improve the system performance and stability of energy harvesting WSANs. In particular, a jointly optimal sampling selection and optimal control policy design by minimizing the infinite-horizon control cost is addressed, and a novel three-step joint optimization algorithm is proposed. The main contributions are summarized as follows.

- In discrete-time domain, the architecture of the WASN system with an energy harvesting controller considering both energy consumption and control strategy design is proposed, and then the WSAN dynamics with network-induced delay is modeled. Based on the analysis of energy harvesting and consumption, the joint optimization problem for energy harvesting WSAN is formulated;
- The joint optimization problem is successfully decomposed into two suboptimal problems. In particular, it can be transformed to be an optimal control strategy design problem for a given sampling interval, while it can be equivalent to an adaptive sampling interval design problem when the control strategy is determined;
- A novel three-step joint optimization algorithm is proposed. First, the optimal sampling interval can be obtained based on the desired energy level, harvested, and remaining energy. Then, the control gain can be derived by using a backward iteration. Finally, the optimal control strategy is determined;
- Numerical experiment results based on the UAV formation flight system are provided to verify the effectiveness of the proposed three-step optimization algorithm for energy harvesting WSANs. The system performances with better control stability and lower energy consumption are achieved.

The remainder of this article is organized as follows. We review the related works about WSAN dynamics control and adaptive sampling, and energy optimization for Energy Harvesting WSAN in Section 2. Next, the proposed energy harvesting WSAN model is presented in Section 3. In Section 4, the joint optimization algorithm considering both energy consumption and network-induced latency is formulated, followed by the proposed three-step joint optimization algorithm. Then, the application of the unmanned aerial vehicle (UAV) system is provided to show the effectiveness of the proposed algorithm for energy harvesting WSANs in Section 5. Finally, we conclude this work in Section 6.

## 2. Related Works

### 2.1. WSAN Control and Adaptive Sampling

In recent years, the growing maturity of integrated electronics technology has promoted the further development of WSAN. How to design the optimal control strategy to improve system performance has become a research hotspot. In [15], an optimal control and scheduling design problem over deterministic real-time networks was studied, which minimizes a quadratic cost function in order to evaluate the control performance and the ability of the adaptive scheduling. However, the above study assumes a perfect system in that which the communication delay is completely ignored. A networked control system model is presented in [16] considering network-induced delays through the wireless communication network, and an optimal controller is designed to address the delay compensation. Then, in [17], a linear quadratic optimal control algorithm is proposed for the discrete-time system when long network delays are considered. In [18], a linear quadratic Gaussian control algorithm was proposed in the multi-hop WSAN to address the collaborative optimization design of control routing and scheduling under energy constraints. Currently, the joint optimization design for the system cost and plant control was investigated in [19] to both reduce the power consumption and improve the control stability. However, the above works focus on the fixed sampling interval. Actually, the determined sampling interval cannot guarantee the energy usage efficiency for the networked control system in many application scenarios [20,21,22]. In [23], an adaptive sampling algorithm that estimates the optimal sampling frequencies for sensors online was proposed to minimize the energy consumption of the sensors. Two adaptive sampling algorithms were proposed in [24] in order to increase the lifetime of WSN by using an optimal sampling rate for monitoring. In [25], the authors provide an energy-aware adaptive sampling algorithm for WSN with power-hungry sensors and harvesting capabilities, an energy management technique that can be implemented on any WSN platform with enough processing power to execute the proposed algorithm. In [26], the authors investigated the variable sampling method to mitigate the effects of time delays in wireless networked control systems using an observer-based control system model. In [27], in order to improve the performance of the networked control system, a variable sampling period scheduling method for the networked control system under resource constraints was presented based on the network operation state.

### 2.2. Energy Optimization for Energy Harvesting WSAN

In recent years, much progress has been made in understanding how to use energy harvesting technology in networking and communications applications [28,29,30,31]. However, there is few works detailing how energy harvesting sensors can be used in control applications, where the closed-loop system’s dynamical behavior is significant. In [32], in order to achieve the energy-neutral operation and system performance improvement, a linear quadratic tracking problem was used to minimize the loss function thus that the duty-cycle computed maintains the specific battery level while all harvested energy was optimally used. In [33], the authors proposed a greedy battery management policy to suffice the plant stability and demonstrate that the optimal control design can be examined by a linear program. However, most current works focus on the battery management of WSN with sensors powered by energy harvesting, and then the joint design of energy management and control strategy in WSAN with energy harvesting capacity is beginning to attract researchers’ attention. In [34], an optimal linear quadratic gaussian control problem with feedback coming from an energy-harvesting sensor was studied. In [35], the optimal LQG controller was obtained by solving the Bellman dynamic programming equation, and a Q-learning algorithm was used to approximate the optimal energy allocation policy in case the system parameters were unknown. A closed-form dynamic energy harvesting and dynamic MIMO precoding solution were proposed for networked control systems with energy harvesting sensors in [36]. Different from energy harvesting sensor nodes, a scenario-based model predictive control approach was exploited to stabilize the plant’s state with the actuator powered by harvested energy in [37].

Unfortunately, there is seldom literature considering controllers with energy harvesting functions in WSAN. In addition, most existing optimal control algorithms focus on the perfect traffic system that the communication delays are ignored. In this paper, considering the network-induced delay as well as the transmission energy consumption of the communication network with an energy harvesting controller, the optimal control strategy design and adaptive sampling selection policy for WSAN are addressed.

## 3. Energy Harvesting WSAN Modeling

**Figure 1:**
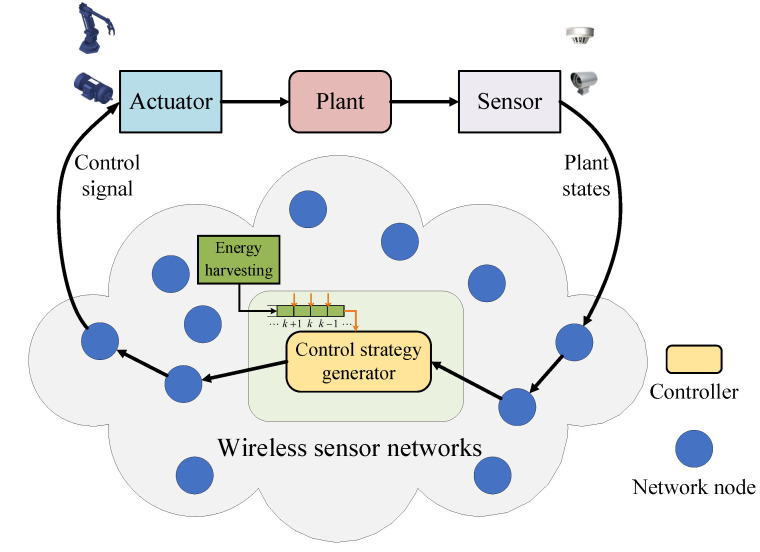
The architecture of WASN system with energy harvesting controller.

As shown in Figure 1, a typical WSAN, consisting of the controller, plant, actuator, and a number of sensors connected through a shared wireless network is considered [34,35]. In particular, compared with the traditional controller powered by non-rechargeable batteries, a controller with the capability of energy harvesting was considered, in which the energy harvesting devices such as solar panels and micro wind turbines were equipped thus that the controller energy can be harvested from the surrounding environment.

In energy harvesting WSAN, the plant states can be periodically sampled and transmitted to the controller through the shared wireless communication network. Once the sampling state information is received, the controller immediately calculates the control strategy and then forwards it to the actuator. Finally, the actuator executes the control signal to ensure the dynamic stability of the plant. During the closed-loop control, the controller energy will be continuously consumed for information reception, storage, calculation, transmission, etc. At the same time, the energy of the controller is supplemented by energy harvesting, thus as to achieve energy consumption balance. In the energy harvesting WSAN, some typical key assumptions are also used [38]: (1) the battery capacity of the energy harvesting in the controller is assumed to be infinite. This is because the capacity of even a small button battery is usually sufficient for energy harvesting scenarios; (2) the energy may be harvested at any time, but the harvested energy can only be used from the next control frame.

### 3.1. WSAN Dynamics Model

In the control process of WSAN, due to the shared wireless network, the effect of network-induced delays cannot be ignored, which will result in a significant system performance degradation or even a system crash. The network-induced delay is mainly introduced by the sensor-to-controller delay, signal processing time, and controller-to-actuator delay. Therefore, the dynamics model for WSAN in a continuous-time domain can be expressed as [19]

(1) $$\dot{s}\left(t\right)=As\left(t\right)+Bc\left(t-\tau \right),$$

where $s\left(t\right)$ is the *K*-dimensional state vector, which is typically defined as the plant state error, $c\left(t\right)$ is the *N*-dimensional control signal vector, *A* and *B* are determined system parameters, and τ is the network-induced delay, which is typically assumed to be smaller than one sampling interval.

Then, the corresponding discrete-time dynamics in i-th sampling interval is given by [16]

(2) $$s_{i+1}=A_{0}s_{i}+B_{1}c_{i}+B_{2}c_{i-1},$$

where

$$\begin{array}{l} s_{i}=s\left(i\Delta T\right),\;c_{i}=c\left(i\Delta T\right),\;A_{0}{=e}^{A\Delta T},\\ B_{1}=\int_{0}^{\Delta T-\tau }e^{A\Delta T}dtB,\;B_{2}=\int_{\Delta T-\tau }^{T}e^{A\Delta T}dtB, \end{array}$$

and ΔT denotes the sampling interval.

The objective of the optimal control strategy design is to ensure the stability of WSAN through minimizing the normalized cost function, which is typically defined as a normalized quadratic form as [35]

(3) $$J_{WSAN}=\frac{1}{M}\left[s_{M}^{T}Rs_{M}+\sum_{i=0}^{M-1}\left(s_{i}^{T}Rs_{i}+c_{i}^{T}Qc_{i}\right)\right],$$

where *R* and *Q* are determined system parameters, and *M* is the finite time horizon.

### 3.2. Energy Harvesting and Consumption

**Figure 2:**
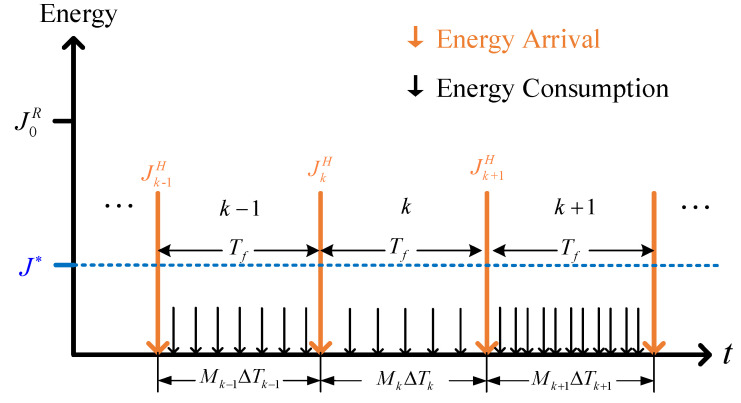
Energy harvesting and consumption model for the controller.

In this subsection, we will describe the energy harvesting and consumption model of how the controller collects, stores, and consumes energy. As shown in Figure 2, the energy arrival may occur at any time, but the harvested energy can only be released at the beginning of the next control frame, which includes $M_{k}$ sampling intervals at the *k*-th control frame. While the controller consumes energy due to signal processing and transmission in each sampling interval. In addition, the battery capacity is usually assumed to be infinity because even a small button battery has enough energy capacity to meet the needs of most energy harvesting schemes [38]. The objective of energy harvesting and consumption is to try to improve the system stability based on the joint design of control strategy and adaptive sampling interval through the effective use of harvesting energy. In general, the energy consumption of the controller in the *k*-th control frame is mainly determined by the signal transmission, which is given by [11]

(4) $$J_{k}^{C}=\sum_{i=0}^{M_{k}-1}\left(\mu +\lambda d^{r}\right)=M_{k}\left(\mu +\lambda d^{r}\right),$$

where $J_{k}^{C}$ denotes the energy consumption for the signal transmission from the controller to the next network node, $M_{k}=T_{f}/\Delta T_{k}$ denotes the number of sampling intervals in the *k*-th control frame, *d* is the transmission distances, $r\in \left[2,4\right]$ is the signal attenuation factor, λ and μ are determined parameters by path loss and signal amplitude, respectively.

Define $J_{k}^{H}$ and $J_{k}^{R}$ as the harvested energy and remaining energy of the *k*-th control frame, respectively. Then, the evolution of the remaining energy in the controller can be modeled as

(5) $$J_{k+1}^{R}=J_{k}^{R}+J_{k}^{H}-J_{k}^{C}.$$

In order to make full use of the energy of the controller, the remaining energy of controller is expected to be maintained at the desired level $J_{k}^{*}$ that

(6) $$J_{k+1}^{R}\to J_{k}^{*}.$$

## 4. Joint Optimization Algorithm Design

In this section, the joint optimization problem for energy harvesting WSAN is formulated. Then, a three-step optimal algorithm is proposed to jointly design the control strategy and adaptive sampling interval.

### 4.1. Joint Optimization Problem

Based on (3) and (6), the utility function of joint optimization problem in *k*-th control frame can be defined as a weighted cost function.

(7) $$J_{joint}=\beta J_{WSAN}+\gamma \left|J_{k+1}^{R}-J_{k}^{*}\right|.$$

where β and γ are weight coefficients.

The objective of the joint optimization is to minimize the utility function subject to system dynamics and the evolution of remaining energy through the designs of both control strategy and adaptive sampling interval. Therefore, the joint optimization problem of the *k*-th control frame can be modeled as

(8) $$\begin{array}{l} \underset{\left\{M_{k},c_{i,k},\forall i\right\}}{\min}\;\beta J_{WSAN}+\gamma \left|J_{k+1}^{R}-J_{k}^{*}\right|\\ s.t.\;s_{i+1,k}=A_{0}s_{i,l}+B_{1}c_{i,k}+B_{2}c_{i-1,k}. \end{array}$$

Actually, at each control frame, the harvested energy will be released at the beginning of the control frame, and then the controller gradually consumes energy in each sampling interval. The remaining energy is continuously decreasing along with the controller’s energy consumption. In other words, the remaining energy is always larger than the desired energy level $J^{*}$ in a control frame. Therefore, the joint optimization problem in (8) can be equivalent to

(9) $$\begin{array}{l} \underset{\left\{M_{k},c_{i,k},\forall i\right\}}{\min}\;\beta J_{WSAN}+\gamma \left(J_{k+1}^{R}-J_{k}^{*}\right)\\ s.t.\;s_{i+1,k}=A_{0}s_{i,l}+B_{1}c_{i,k}+B_{2}c_{i-1,k},\\ J_{k+1}^{R}\geq J^{*}. \end{array}$$

The optimization problem (9) is a typical NP hard problem, which is difficult to directly solve it. Fortunately, it can decompose the joint optimization problem into two suboptimal problems: (1) for a given sampling interval $T_{f}/M_{k}^{*}$, it can be transformed to be an optimal control strategy design problem; (2) when the control strategy $\left\{c_{i,k}^{*}\right\}$ is determined, it can be equivalent to be a subproblem to address the adaptive sampling interval design. That is

(10) $$\begin{array}{l} S1:\;\underset{\left\{c_{i,k}\right\}}{\min}\;s_{M_{k}^{*},k}^{T}\bar{R}s_{M_{k}^{*},k}+\sum_{i=0}^{M_{k}^{*}-1}\left(s_{i,k}^{T}\bar{R}s_{i,k}+c_{i,k}^{T}\bar{Q}c_{i,k}\right)\\ s.t.\;s_{i+1,k}=A_{0}s_{i,k}+B_{1}c_{i,k}+B_{2}c_{i-1,k}. \end{array}$$

(11) $$\begin{array}{l} S2:\;\underset{M_{k}}{\min}\;J_{k}^{R}+J_{k}^{H}-J_{k}^{*}-M_{k}\left(\mu +\lambda d^{r}\right)\\ s.t.\;J_{k}^{R}+J_{k}^{H}-J_{k}^{*}-M_{k}\left(\mu +\lambda d^{r}\right)\geq 0. \end{array}$$

where

$$\bar{R}=\frac{\beta R}{M_{k}},\;\bar{Q}=\frac{\beta Q}{M_{k}}-\mu -\lambda d^{r}.$$

In general, the control strategy and sampling interval should be calculated by subproblems *S*1 and *S*2, respectively, and then iteratively converge to the joint optimization results. However, the iteration process always has extremely large computational complexity. Fortunately, it was found that the relationship between the adaptive sampling interval selection and optimal control strategy design can be totally decoupled. For a given sampling interval, the optimal corresponding control strategy can be firstly derived as a function of the given sampling interval. Then, the optimal selection of the sampling interval can be determined by the energy harvesting, consumption, and remaining level requirements.

### 4.2. Control Strategy Design

We first address the optimal control strategy design problem (10) subject to a given sampling interval.

Define

(12) $${\tilde{s}}_{i,k}=\left[\begin{array}{c} s_{i,k}\\ c_{i-1,k} \end{array}\right].$$

Then, the discrete-time dynamics can be rewritten as

(13) $${\tilde{s}}_{i+1,k}=\tilde{A}{\tilde{s}}_{i,k}+\tilde{B}c_{i,k},$$

where

$$\tilde{A}=\left[\begin{array}{cc} A_{0} & B_{2}\\ 0_{N\times K} & 0_{N\times N} \end{array}\right],\;\tilde{B}=\left[\begin{array}{c} B_{1}\\ I_{K\times K} \end{array}\right].$$

and $0_{i\times j}$ and $I_{i\times i}$ denote the i×j zero matrix and i×i identity matrix, respectively.

By using the new state vector ${\tilde{s}}_{i,k}$, the joint optimization problem (10) can be equivalent to the following problem

(14) $$\begin{array}{l} \underset{\left\{c_{i,k}\right\}}{\min}\;{\tilde{s}}_{M_{k}^{*},k}^{T}\tilde{R}{\tilde{s}}_{M_{k}^{*},k}+\sum_{i=0}^{M_{k}^{*}-1}\left({\tilde{s}}_{i,k}^{T}\tilde{R}{\tilde{s}}_{i,k}+c_{i,k}^{T}\bar{Q}c_{i,k}\right)\\ s.t.\;{\tilde{s}}_{i+1,k}=\tilde{A}{\tilde{s}}_{i,k}+\tilde{B}c_{i,k}, \end{array}$$

where

$$\tilde{R}=\left[\begin{array}{cc} \bar{R} & 0_{K\times N}\\ 0_{N\times K} & 0_{N\times N} \end{array}\right].$$

Define the residual cost as

(15) $$J_{i,k}^{Re}=\underset{\left\{c_{j,k}\right\}}{\min}\;\left[{\tilde{s}}_{M_{k}^{*},k}^{T}\tilde{R}{\tilde{s}}_{M_{k}^{*},k}+\sum_{j=i}^{M_{k}^{*}-1}\left({\tilde{s}}_{i,k}^{T}\tilde{R}{\tilde{s}}_{i,k}+c_{i,k}^{T}\bar{Q}c_{i,k}\right)\right].$$

**Theorem** **1.** *The optimal control strategy design for (14) is given by*

(16) $$c_{i,k}^{*}=-g_{i,k}{\tilde{s}}_{i,k},\;i=0,\;1,\ldots ,\;M_{k}^{*}-1,$$

*where* $g_{i,k}$*can be iteratively calculated as*

(17) $$\begin{array}{l} g_{i,k}={\left[{\tilde{B}}^{T}l_{i,k+1}\tilde{B}+\bar{Q}\right]}^{-1}{\tilde{B}}^{T}l_{i,k+1}\tilde{A},\\ l_{i,k}={\tilde{A}}^{T}l_{i,k+1}\tilde{A}+\tilde{R}-g_{ik}^{T}{\tilde{B}}^{T}l_{i,k+1}\tilde{A},\\ l_{M_{k}^{*},k}=\tilde{R}, \end{array}$$

*and the corresponding residual cost in (15) can be derived in a quadratic form as*

(18) $$J_{i,k}^{Re}={\tilde{s}}_{i,k}^{T}l_{i,k}{\tilde{s}}_{i,k}.$$

**Proof.**  The optimal control strategy can be deduced by a backward recursion approach.

Assuming $J_{j,k}^{Re},\;j>i$ has the same quadratic form as (18) that

(19) $$J_{j,k}^{Re}={\tilde{s}}_{j,k}^{T}l_{j,k}{\tilde{s}}_{j,k}.$$

Then, the residual cost function $J_{i,k}^{Re}$ given as follows


(20) $$\begin{array}{cc} J_{i,k}^{Re} & =\min\left\{{\left[\begin{array}{c} {\tilde{s}}_{i,k}\\ c_{i,k} \end{array}\right]}^{T}\left[\begin{array}{cc} \tilde{R} & 0\\ 0 & \bar{Q} \end{array}\right]\left[\begin{array}{c} {\tilde{s}}_{i,k}\\ c_{i,k} \end{array}\right]\right\}+J_{i+1,k}^{Re}\\ & =\min\left\{{\left[\begin{array}{c} {\tilde{s}}_{i,k}\\ c_{i,k} \end{array}\right]}^{T}\left[\begin{array}{cc} \tilde{R} & 0\\ 0 & \bar{Q} \end{array}\right]\left[\begin{array}{c} {\tilde{s}}_{i,k}\\ c_{i,k} \end{array}\right]\right\}+{\tilde{s}}_{j+1,k}^{T}l_{j+1,k}{\tilde{s}}_{j+1,k}\\ & =\min\left\{{\left[\begin{array}{c} {\tilde{s}}_{i,k}\\ c_{i,k} \end{array}\right]}^{T}\left[\begin{array}{cc} e_{i,k}^{1,1} & {\left(e_{i,k}^{2,1}\right)}^{T}\\ e_{i,k}^{2,1} & e_{i,k}^{2,2} \end{array}\right]\left[\begin{array}{c} {\tilde{s}}_{i,k}\\ c_{i,k} \end{array}\right]\right\} \end{array}$$

where


$$\begin{array}{cc} e_{i,k}^{1,1} & ={\tilde{A}}^{T}l_{i,k+1}\tilde{A}+\tilde{R},\\ e_{i,k}^{2,2} & ={\tilde{B}}^{T}l_{i,k+1}\tilde{B}+\bar{Q},\\ e_{i,k}^{2,1} & ={\tilde{B}}^{T}l_{i,k+1}\tilde{A}, \end{array}$$

It can be seen that $J_{i,k}^{Re}$ is a quadratic form of $c_{i,k}$. In order to derive the minimum value for the $J_{i,k}^{Re}$ based on (15) and (20), the optimal control strategy can be deduced as 


(21) $$\begin{array}{ll} c_{i,k}^{*} & =\underset{\left\{c_{i,k}\right\}}{\arg}\min\left\{J_{i,k}^{Re}\right\}\\ & =-g_{i,k}{\tilde{s}}_{i,k}, \end{array}$$

where


(22) $$\begin{array}{ll} g_{i,k} & =-{\left(e_{i,k}^{2,2}\right)}^{-1}e_{i,k}^{2,1}\\ & ={\left[{\tilde{B}}^{T}l_{i,k+1}\tilde{B}+\bar{Q}\right]}^{-1}{\tilde{B}}^{T}l_{i,k+1}\tilde{A}. \end{array}$$

and the corresponding residual cost function can be derived in the quadratic form as in (18). □

Thus, it can be seen that the optimal control strategy $c_{i,k}^{*}$ can be obtained on-line by a linear function of current plant states and previous control signals given by (16), in which the corresponding control gain $g_{i,k}$ is derived offline by using backwards iteration based on (17).

### 4.3. Adaptive Sampling Interval Design

Once the optimal control strategy is determined, the joint optimization problem (9) can be simplified to be the adaptive sampling interval design problem as

(23) $$\underset{M_{k}}{\min}\;\left|J_{k}^{R}+J_{k}^{H}-J_{k}^{*}-M_{k}\left(\mu +\lambda d^{r}\right)\right|.$$

Actually, at each control frame, the harvested energy will be released at the beginning of the control frame, and then the controller gradually consumes energy in each sampling interval. The remaining energy is continuously decreasing along with the controller’s energy consumption. In other words, the remaining energy is always larger than the desired energy level $J^{*}$ in a control frame. Therefore, the adaptive sampling interval design problem (23) is equivalent to

(24) $$\underset{M_{k}}{\min}\;J_{k}^{R}+J_{k}^{H}-J_{k}^{*}-M_{k}\left(\mu +\lambda d^{r}\right).$$

Then, the optimal number of sampling intervals can be derived when the remaining energy is equal to the desired energy level at the end of the control frame. That is

(25) $$\begin{array}{l} J_{k}^{R}+J_{k}^{H}-J^{*}-M_{k}^{*}\left(\mu +\lambda d^{r}\right)=0\\ \Leftrightarrow \frac{T_{f}}{\Delta T_{k}^{*}}=\frac{J_{k}^{R}+J_{k}^{H}-J_{k}^{*}}{\mu +\lambda d^{r}} \end{array}$$

Based on (25), the optimal sampling interval is given by

(26) $$\Delta T_{k}^{*}=\frac{T_{f}\left(\mu +\lambda d^{r}\right)}{J_{k}^{R}+J_{k}^{H}-J_{k}^{*}}.$$

Thus, the joint optimization design of the energy consumption and control strategy for energy harvesting wireless sensor networks can be summarized as in Algorithm 1 by a three-step procedure below. Firstly, the adaptive sampling interval design $\Delta T_{k}^{*}$ can be determined by (25) based on the harvested energy, remaining energy of the last control frame, desired energy level, and transmission environments. Then, the optimal control gain $\left\{g_{i,k}\right\}$ is iteratively calculated off-line by (17). Finally, the optimal control strategy $\left\{c_{i,k}^{*}\right\}$ can be derived by (16) in real-time for each sampling interval based on the current plant states, optimal control gain, and previous control signals. 

**Algorithm 1** Three-Step Algorithm 1    **Step 1: Off-line**2    Set System paramters *μ*, *λ*, *d*, *r* and $J_{k}^{*}$.3    Update initializations $J_{k}^{R}$ and $J_{k}^{H}$
4    Calculate the optimal sampling interval $\Delta T_{k}^{*}=T_{f}\left(\mu +\lambda d^{r}\right)/J_{k}^{R}+J_{k}^{H}-J_{k}^{*}.$
5    **Step 2: Off-line**6    Initialize $l_{M_{k}^{*},k}=\tilde{R}$7    **for**
$i=M_{k}^{*}-1:-1:0$ **do**8      Calculate $l_{i,k}={\tilde{A}}^{T}l_{i,k+1}\tilde{A}+\tilde{R}-g_{ik}^{T}{\tilde{B}}^{T}l_{i,k+1}\tilde{A}$.9      Calculate $g_{i,k}={\left[{\tilde{B}}^{T}l_{i,k+1}\tilde{B}+\bar{Q}\right]}^{-1}{\tilde{B}}^{T}l_{i,k+1}\tilde{A}$.10    **end**11    **Step 3: On-line**12    Initialize $s_{0,k}$$,\;c_{l,k}^{*}=0,\;l\leq 0$.13    **for**
$i=0:1:M_{k}^{*}-1$ **do**14      Update plant states $s_{i,k}$.15      Set ${\tilde{s}}_{i,k}={\left[s_{i,k}^{T},c_{i-1,k}^{*}\right]}^{T}$.
16      Calculate the optimal control $c_{i,k}^{*}=-g_{i,k}{\tilde{s}}_{i,k}$.
17    **end**

## 5. Simulations and Discussion

**Figure 3:**
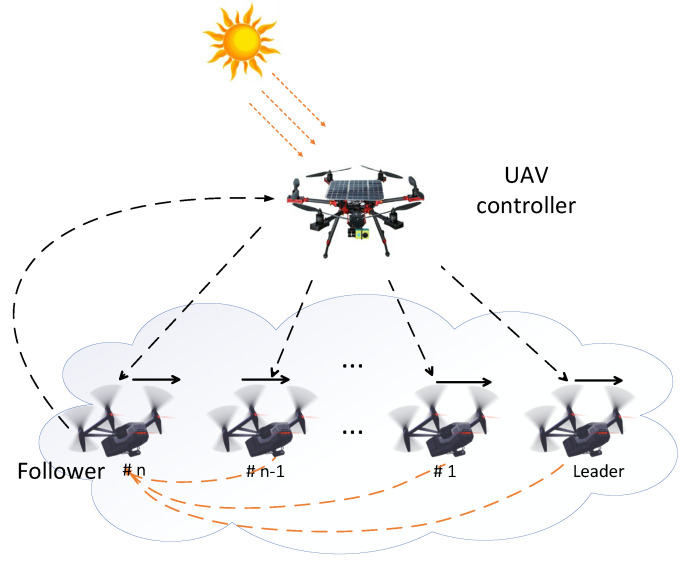
The UAV formation flight system with an energy harvesting controller.

The application of the UAV formation flight system with an energy harvesting controller is provided to show the effectiveness of the proposed three-step optimization algorithm for WSANs. The UAV formation flight system, including a solar-powered UAV controller, a UAV leader, and multiple UAV followers, is shown in Figure 3. The UAV controller collects the position and speed information of the leader. Once the UAV controller receives the state information of the leader, it immediately calculates the control strategy and selects the optimal sampling period according to the situation of solar energy charging and energy consumption in order to maintain the UAV formation flight system stably and efficiently. As a case study, a typical three-UAV platoon traveling on a horizontal path is considered; the UAV formation flight system has one UAV follower, one leader, and a solar-powered UAV controller. The states of UAV formation flight system are given by

(27) $$s\left(t\right)={\left[h\left(t\right),\;v\left(t\right)\right]}^{T},$$

where $h\left(t\right)$ and $v\left(t\right)$ represent the UAV follower’s position error and speed error, respectively.

The purpose of UAV formation flight control is to maintain the formation of the follower when the UAV state is disturbed by the external environments, such as wind and state noises. That is, the control signal is to ensure the state deviation remains within a limited range. In the simulations, the initialization position and velocity errors are set to be zero, which is disturbed by the random noise. The control frame is set as $T_{f}=5\left[s\right]$, the fixed sampling interval is set as 0.083[s], the initial energy of UAV controller is $J_{0}^{R}=20$, the desired energy level $J^{*}=10$, the minimum energy level $J_{\min}=5$, and the system parameters are set as follows.

(28) $$\begin{array}{l} A=\left[\begin{array}{cc} 0 & 1\\ 0 & 0 \end{array}\right],\;B=\left[\begin{array}{c} 0\\ 1 \end{array}\right],\\ R=\left[\begin{array}{cc} 1 & 0\\ 0 & 1 \end{array}\right],\;Q=1. \end{array}$$

In order to demonstrate the effectiveness of the proposed algorithm, three energy harvesting cases, including poor, sufficient, and general energy harvesting conditions, are considered, and the performance comparisons with the existing work [19] with traditional fixed sampling interval are shown.

**Figure 4:**
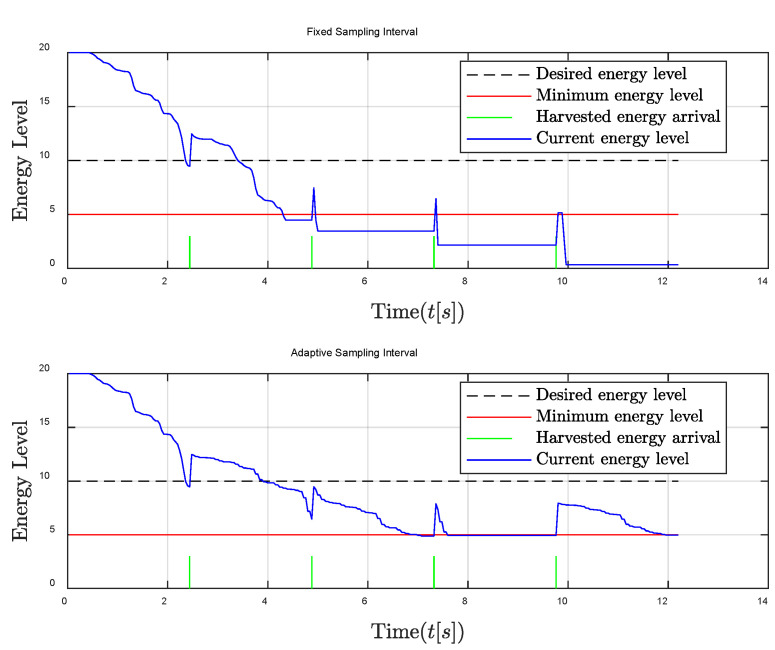
Energy level comparison between fixed and adaptive sampling intervals in the poor energy harvesting condition.

**Figure 5:**
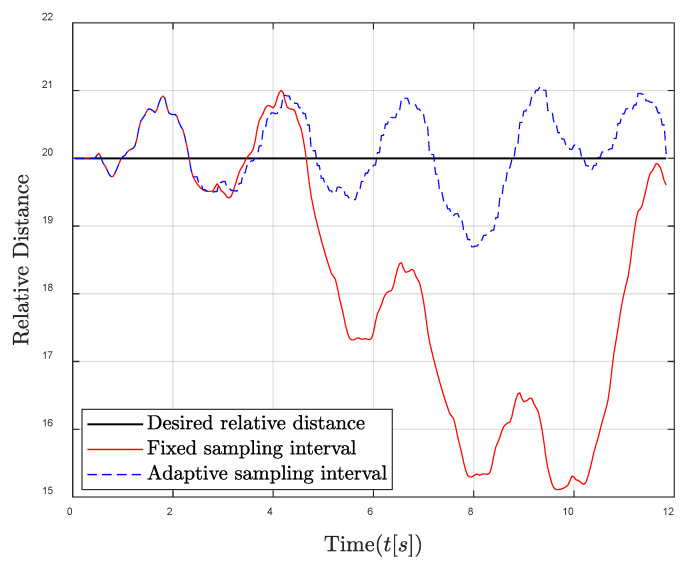
The relative distance between the follower and the leader comparisons in the poor energy harvesting condition.

First, the poor energy harvesting condition such as cloudy weather, where the harvested energy is not enough, is investigated. As seen in Figure 4, the energy of the controller using the traditional fixed sampling method decreases rapidly and then suddenly drops below the minimum energy level, which will cause the controller to fail to work. This is because the fixed sampling interval causes more energy to be consumed than harvested, thus that the remaining energy level gradually decreases and may even exhaust the remaining energy to make the control system shut down. Compared with the fixed sampling interval, the energy of the controller using the adaptive sampling interval is also difficult to keep at the expected value due to insufficient energy harvested, but the energy of the controller can still be higher than the minimum energy level to maintain the normal work of the system. This is because the sampling interval is automatically adjusted to become larger to save energy when the remaining energy level is low. The control performance comparison is shown in Figure 5; it can be seen that a significant performance improvement is achieved compared to that of the fixed sampling interval. Especially when the energy level falls below the minimum energy level, the controller cannot work properly; thus that severe control stability degradation is caused in the case of fixed sampling interval.

**Figure 6:**
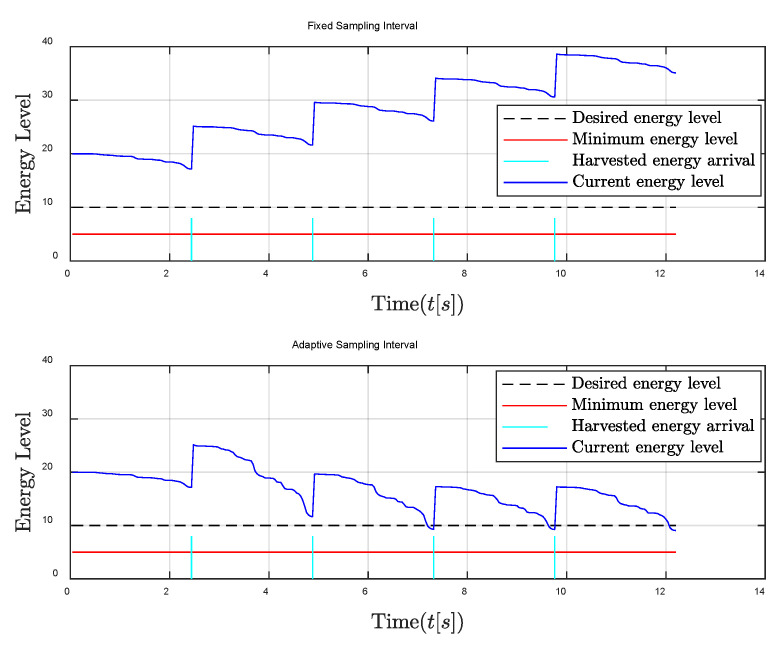
Energy level comparison between fixed and adaptive sampling intervals in the sufficient energy harvesting condition.

**Figure 7:**
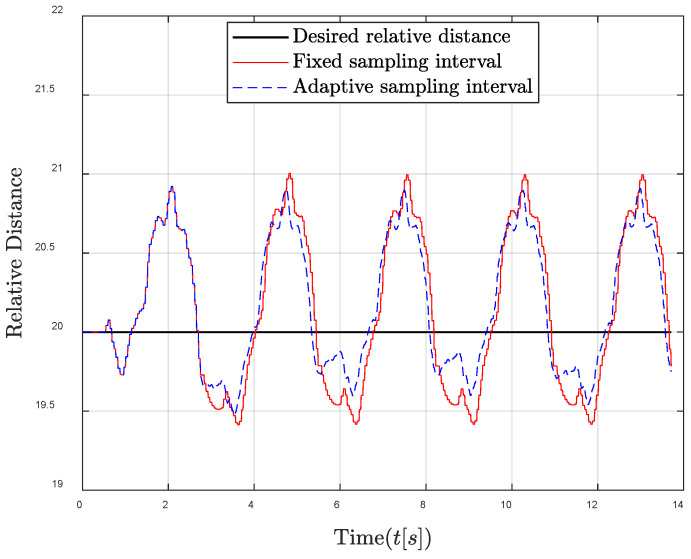
Relative distance between the follower and the leader comparisons in the sufficient energy harvesting condition.

Then, the performances of the proposed algorithm in sufficient energy harvesting conditions are shown in Figure 6 and Figure 7. It can be seen that the remaining energy of the traditional fixed sampling interval gradually increases. This is because the remaining energy cannot be effectively utilized in sufficient energy harvesting conditions due to the fixed sampling interval, and the harvested energy is always greater than the consumed energy in each sampling interval. Fortunately, through the adaptive sampling interval algorithm, the controller energy can be maintained near the required energy level; thus that the remaining energy and harvested energy in each control frame can be fully used to improve the system control performance. Similarly, Figure 7 also shows that the oscillation reduction of the relative distance between the follower and the leader can be achieved by the adaptive sampling interval strategy, especially when the oscillation of the relative distance is large.

**Figure 8:**
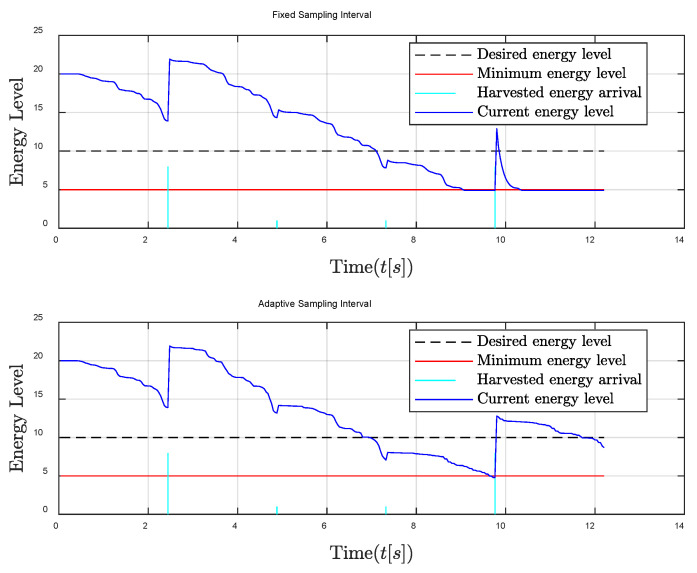
Energy level comparison between fixed and adaptive sampling intervals in the general energy harvesting condition.

**Figure 9:**
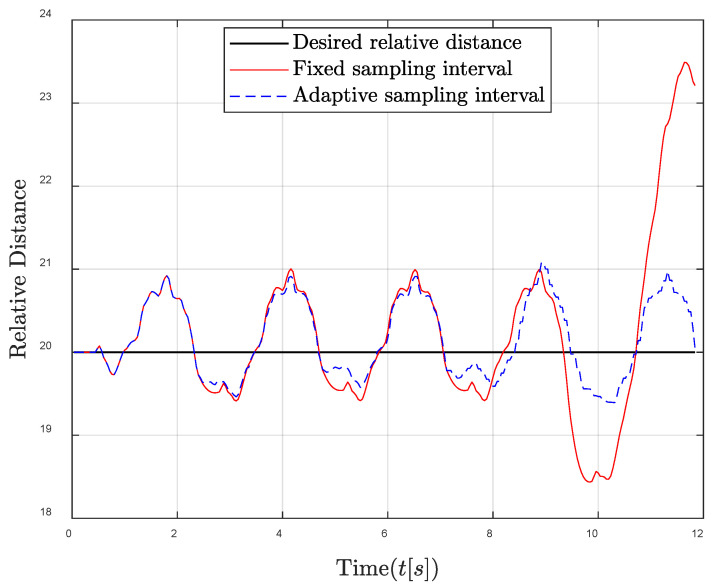
Relative distance between the follower and the leader comparisons in the general energy harvesting condition.

Finally, the general energy harvesting condition is considered in Figure 8 and Figure 9. It can be observed that the performance of the adaptive sampling interval is slightly better when the remaining energy level is high, which is similar to the case of sufficient energy harvesting conditions. While when the remaining energy level is low, the traditional fixed sampling period will suffer significant performance degradation, which is similar to the poor energy harvesting condition.

To sum up, the proposed joint optimization design of control strategy and energy consumption can guarantee the system performance and control stability for all poor, sufficient, and general energy harvesting conditions. Compared to the traditional fixed sampling interval approach, the proposed joint optimization algorithm can successfully avoid the serious control instability when the remaining energy level is low and can also efficiently use up the harvested energy when the remaining energy level is high.

## 6. Conclusions

In this paper, the joint optimization algorithm of physical plant control, energy harvesting, and energy consumption toward the WSAN system is proposed when the network-induced delays caused by wireless communications are considered. The architecture of the WASN system with an energy harvesting controller considering both energy consumption and control strategy design is modeled, and then the joint optimization problem is formulated based on the collaborative utility function and WSAN dynamics. With the objective of minimizing the utility function subject to system dynamics and the evolution of remaining energy, a three-step algorithm is proposed for the closed-loop feedback control. The sampling interval is firstly determined by the information of desired energy level, harvested, and remaining energy. Then, the control gain can be obtained by using a backward iteration. Finally, the optimal control strategy is derived from meeting both requirements of control stability and energy efficiency. A case study of the UAV formation flight system is introduced to demonstrate the effectiveness of the proposed joint optimization design that the serious control instability can be avoided when the remaining energy level is low, while the harvested energy can be efficiently used up when the remaining energy level is high.

## Data Availability

The data presented in this study are available on request from the corresponding author.
